# Characterization of a newly identified rice chitinase-like protein (OsCLP) homologous to xylanase inhibitor

**DOI:** 10.1186/1472-6750-13-4

**Published:** 2013-01-18

**Authors:** Jingni Wu, Yiming Wang, Sun Tae Kim, Sang Gon Kim, Kyu Young Kang

**Affiliations:** 1 Division of Applied Life Science (BK21 program), Gyeongsang National University, Jinju, 660-701, South Korea; 2 Plant Molecular Biology and Biotechnology Research Center, Gyeongsang National University, Jinju, 660-701, South Korea; 3 Department of Plant Bioscience, Pusan National University, Miryang, 627-706, South Korea

**Keywords:** Antifungal activity, Chitinase, Oryza sativa, Xylanase inhibitor

## Abstract

**Background:**

During rice blast fungal attack, plant xylanase inhibitor proteins (XIPs) that inhibit fungal xylanase activity are believed to act as a defensive barrier against fungal pathogens. To understand the role of XIPs in rice, a xylanase inhibitor was cloned from rice. The expression of this gene was examined at the transcriptional/translational levels during compatible and incompatible interactions, and the biochemical activity of this protein was also examined.

**Results:**

Sequence alignment revealed that the deduced amino acid sequence of OsCLP shares a high degree of similarity with that of other plant TAXI-type XIPs. However, recombinant OsCLP did not display inhibitory activity against endo-1,4-β-xylanase enzymes from *Aureobasidium pullulans* (*A. pullulans*) or *Trichoderma viride* (*T. viride*). Instead, an in-gel activity assay revealed strong chitinase activity. The transcription and translation of OsCLP were highly induced when rice was exposed to pathogens in an incompatible interaction. In addition, exogenous treatment with OsCLP affected the growth of the basidiomycete fungus *Rhizoctonia solani* through degradation of the hyphal cell wall. These data suggest that OsCLP, which has chitinase activity, may play an important role in plant defenses against pathogens.

**Conclusions:**

Taken together, our results demonstrate that OsCLP may have antifungal activity. This protein may directly inhibit pathogen growth by degrading fungal cell wall components through chitinase activity.

## Background

Plants have developed survival strategies, including the strengthening of plant cell walls, to protect themselves from continuous abiotic and biotic stresses. As the first battlefield of the plant-pathogen interaction, plant cell walls, which are mainly composed of various polysaccharides such as cellulose and hemicelluloses, provide tensile strength to plant cells and protect them from biotic invasion [1,2].

During infection, plant pathogens secrete numerous cell-wall degradation enzymes (CWDE) such as cellulases (e.g., endo-β-1,4-glucanase), pectinases (e.g., pectin lyase, polygalacturonase), and endo-β-1,4-xylanase to degrade the cell wall and allow the pathogen to enter in the cell. The majority of these CWDEs belong to the glycosyl hydrolase (GH) family, based on similarities in amino acid sequence, catalytic domains, protein folds, and overall architecture [3,4]. Recently, CWDEs have attracted interest because of their utility in biotechnological processes, enhancing processes such as bread making [5] and animal feed production [6], as well as their role as pathogenicity factors in plant pathogenic microbes [7]. The microbial GH endoxylanase is an important enzyme in the hydrolysis of xylans, catalyzing the hydrolysis of β-1,4-glycosidic linkages between the xylofuranosyl units in the xylan main chain in both cereals and hardwoods [8]. Comprehensive functional studies based on structural, biochemical, and molecular properties of these enzymes have been reported [9,10].

At the same time, plants secrete a group of proteinaceous xylanase inhibitors to suppress pathogenic xylanases. These proteins are thought as “defense molecules” that protect plant cells from attack by pathogenic hydrolytic enzymes [11]. Two types of plant xylanase inhibitors have been well studied, i.e., xylanase-inhibiting protein (XIP)-type inhibitors [12] and *Triticum aestivum* xylanase inhibitor (TAXI)-type inhibitors [13,14]. Biochemical analysis revealed that a wheat XIP specifically inhibits the expression of family-10 and −11 xylanases from *Aspergillus nidulans and Aspergillus niger*, respectively [15], while a TAXI inhibits the expression of the family-11 xylanase (but not the family-10 xylanase) of *A. niger* and *Bacillus subtilis*[13,16].

Recently, three rice XIPs, including OsXIP, rice XIP, and a putative rice xylanase inhibitor (RIXI), were found to be differentially expressed during various developmental stages and under stress conditions [17-19]. Among these, OsXIP was predicted to be a class III chitinase, based on bioinformatics analysis; however, no chitinase activity was detected for this protein. Instead, the protein showed dosage-dependent xylanase inhibitor activity [17,20].

In this study, we functionally characterized a chitinase-like protein from rice (OsCLP) that is expressed at both the transcriptional and translational levels during fungal pathogen infection. The recombinant OsCLP protein has strong chitinase activity and can dissolve cell walls, leading to the release of cytosolic contents. This is the first report on TAXI-like molecule with chitinase activity. This work provides new insights into the function of OsCLP in plant defense mechanisms.

## Results and discussion

### Sequence analysis and characterization of OsCLP

**Figure 1:**
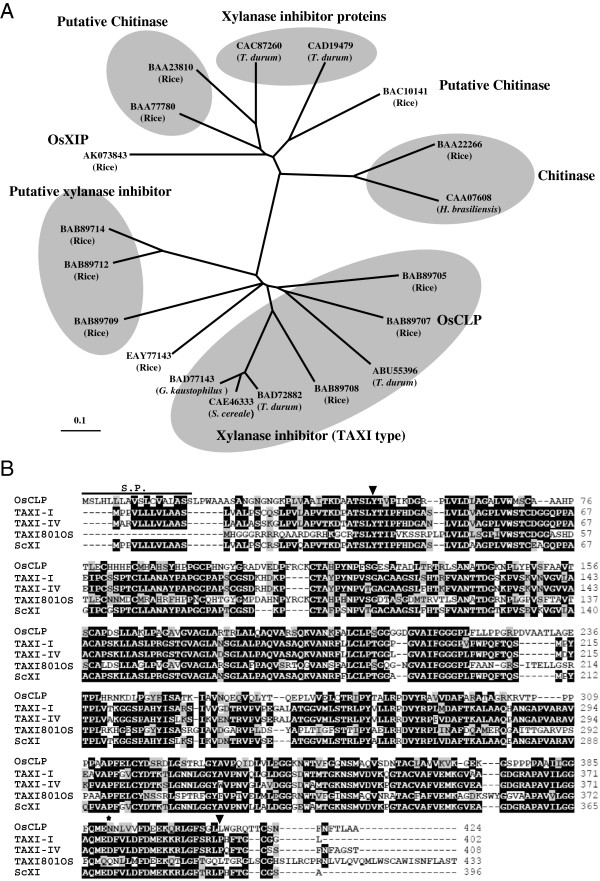
**Analysis of the amino acid sequence of OsCLP. A**, phylogenetic tree for plant chitinases and xylanase inhibitors. All amino acid sequences were retrieved from the National Center for Biotechnology Information (NCBI) database. **B**, amino acid sequences of XIs were obtained from the NCBI data bank (http://www.ncbi.nlm.nih.gov/) with the accession numbers: *Oryza sativa* OsCLP (BAB89707), *Triticum aestivum* TAXI 801OS (ABU55396), *Triticum aestivum* TAXI-IV (BAD72882), *Secale cereal* ScXI (CAE46333), and *Triticum aestivum* TAXI-I (BAD72880). Alignment was done using the BioEdit program and shaded by boxshade. Identical amino acids are shaded in black and similar ones are shaded in gray. The signal peptide region is indicated with lines. The inverted triangles indicate the beginning and end of the xylanase inhibitor I-like domain. The protein-protein interaction site is indicated with a star.

From previous apoplastic secretome analysis of the rice blast fungus interaction (unpublished), an XIP, which was highly expressed upon *Magnaporthe oryzae* (*M. oryzae*) infection, was isolated and identified (data not shown). We then obtained a full-length rice xylanase inhibitor like chitinase gene using PCR. The deduced amino acid sequence of the OsCLP comprises 424 amino acids with an expected molecular mass of 44.6 kDa and a p*I* of 8.51. A homology search of the deduced amino acid sequence of this gene using the GenBank database revealed that OsCLP is homologous to TAXI-type xylanase inhibitor (Figure 1A). OsCLP contains an 18 amino acid signal sequence at the N-terminus and a protein-protein interaction site (Asn 390) at the C-terminus (Figure 1B). This protein contains a putative xylanase inhibitor I-like domain between Tyr48 and Leu408 and is classified as a putative TAXI-type inhibitor. Such inhibitors have similar structures to those of the pepsin-like family of aspartic proteases [21]. However, OsCLP does not contain the catalytic domain GxDxDxE, which is highly conserved in all class III chitinases of plants, bacteria, and fungi [22]. A comparison of the deduced amino acid sequence of OsCLP with that of other TAXI-type inhibitors showed that OsCLP shares 48.4% identity with wheat TAXI80S, 44.1% with wheat TAXI-IV, 42.3% with wheat TAXI-I, and 41.5% with rye TAXI-type xylanase inhibitor.

### Purification of recombinant OsCLP

**Figure 2:**
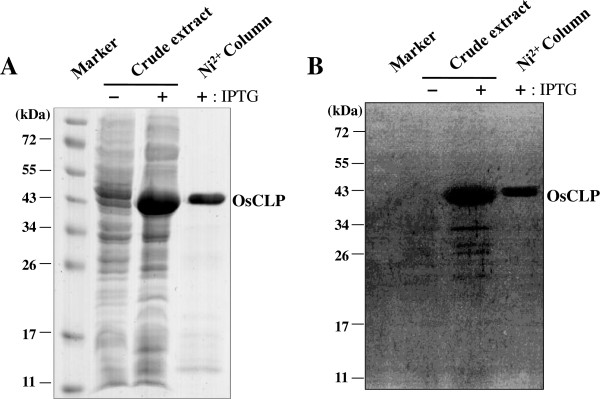
**Purification and in-gel chitinase activity assay of OsCLP. A**, Coomassie blue-stained 12% SDS-PAGE of purified OsCLP. The eluted fractions from the Ni^2+^-affinity resins contained highly purified, soluble OsCLP. A plus sign indicates that IPTG was included in the growth medium. **B**, chitinase activity assay. Protein samples were separated on 12.5% SDS-PAGE gels containing 1% glycol chitin substrate and stained with 0.01% Fluorescent Brightener 28 staining solution.

To measure the biochemical activity of OsCLP, which comprises His-tagged OsCLP without the signal peptide, we attempted to purify recombinant pQE30::OsCLP in *E. coli* using Ni^2+^-affinity resins at various temperatures (37°C, 30°C, 25°C, 18°C, and 4°C) and with various final concentrations of IPTG (1 mM, 0.5 mM, and 0.1 mM). Soluble OsCLP was obtained when cells were cultured at 18°C with IPTG at a final concentration of 0.1 mM. The crude and soluble OsCLP were then separated by 12.5% SDS-PAGE, followed by Coomassie brilliant blue (CBB) staining (Figure 2A). As shown in Figure 2, the protein appeared as one primary band of approximately 44 kDa on SDS-PAGE, which closely matched the calculated MW of 44.6 kDa that was determined after purification of the protein on the Ni^2+^-affinity resins. The purification of soluble recombinant protein made it possible to investigate the biochemical activity of OsCLP.

### Chitinase activity of OsCLP

**Figure 3:**
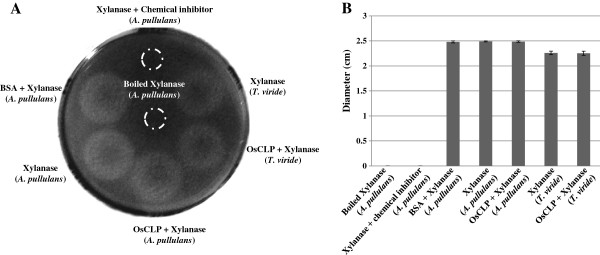
**Xylanase inhibition assay of OsCLP. A**, xylanase inhibition by OsCLP on agar plate containing xylan, as evidenced by the presence of a xylan degradation circle. Inhibition of xylanases isolated from *A. pullulans* and *T. viride* by OsCLP were performed on a 1.5% LB agar plate containing 1% (w/v) birchwood xylan substrate. **B**, analysis of the degree of xylanase inhibition by OsCLP. Samples included 10 μmol boiled xylanase (*A. pullulans*), 10 μmol xylanase (*A. pullulans*) with chemical inhibitor (15 mM *N*-bromosuccinimide), 5 μg BSA with 10 μmol xylanase (*T. viride*), 10 μmol xylanase (*T. viride*) only, 10 μg OsCLP with 10 μmol xylanase (*T. viride*), 10 μmol xylanase (*A. pullulans*) only, and 10 μg OsCLP with 10 μmol xylanase (*A. pullulans*). All statistical analyses were performed using Student’s t test (*P* < 0.05). Dashed circles indicate positions of paper disks.

Bioinformatics analysis revealed that OsCLP is a putative XIP. To confirm the xylanase inhibitor activity of OsCLP, we carried out a xylanase inhibition assay using standard fungal endo-1,4-β-xylanases isolated from *T. aviride* and *A. pullulans*. Ten micrograms of purified OsCLP protein was pipetted onto filter discs, along with 10 μmol of endo-1,4-β-xylanases, on an agar plate containing xylan substrate. Interestingly, OsCLP did not inhibit xylan degradation, while treatment with xylanase inhibitor (15 mM *N*-bromosuccinimide) or boiled xylanase had no activity (Figure 3). Previous research has revealed that XIP-I and TAXI-I can reduce the activity of xylanase, while XIP-I did not show chitinolytic activity due to the presence of salt bridges [23]. However, our results show that OsCLP does not possess xylanase inhibitor activity against endo-1,4-β-xylanases (Figure 3).

Recently, it was reported that an XIP identified from coffee, CaclXIP, plays an important role in the inhibition of Asian soybean rust spore germination through the xylanase inhibitor activity, but not the chitinase activity, of this XIP [24]. Like CaclXIP, OsXIP also has xylanase inhibitor activity, although OsXIP was predicted to be a class III chitinase [17]. In contrast to CaclXIP and OsXIP, which lack chitinase activity, we wanted to confirm that OsCLP has chitinase activity by performing an in-gel chitinase activity assay. In this procedure, crude and purified OsCLP induced by IPTG in *E. coli*, as well as a control with IPTG, were separated by 12.5% SDS-PAGE in a gel containing glycol chitin (Figure 2B). As shown in Figure 2B, strong chitinase activity, in the position in which OsCLP was expressed (Figure 2A), was detected under a UV filter. This suggests that the OsCLP protein displays strong chitinase activity. However, further functional analysis of OsCLP will be required, using biochemical and structural analysis approaches, to understand the different roles played by OsCLP and TAXI-type inhibitors.

### Transcriptional and translational levels of OsCLP after fungal infection

**Figure 4:**
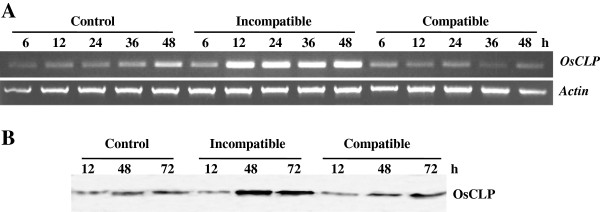
**Transcriptional and translational analysis of *OsCLP *expression in response to incompatible and compatible interactions.** Total RNAs and protein were extracted from rice leaves sprayed with water (control) and inoculated with incompatible (KJ401) or compatible (KJ301) rice blast fungus, *M. oryzae*, at the indicated time point. **A**, transcriptional level of *OsCLP* was analyzed by RT-PCR. Actin was used as a loading control. **B**, translational level of OsCLP was detected by Western blotting.

In previous studies, TAXI-type xylanase inhibitor genes were found to be induced by pathogens in wheat [25]. To determine more accurately the induction level of *OsCLP* in response to infection by rice blast fungus (*M. oryzae*), we evaluated the transcriptional and translational levels of this gene using semi-quantitative reverse transcription PCR (RT-PCR) and Western blot analysis. RT-PCR analysis revealed that the level of *OsCLP* transcript rapidly increased within 12 h of fungal inoculation in the incompatible (Resistance, KJ401) interaction, which displayed few lesion-type symptoms on rice leaves compared to the control and compatible (Susceptible, KJ301) interactions (Figure 4A). At the protein level, OsCLP was shown to be highly and rapidly accumulated in the incompatible interaction at 48 h, while the level of OsCLP slowly increased in the compatible interaction at 72 h (Figure 4B). These results suggest that OsCLP plays an important role in plant defense response.

### Antifungal activity of OsCLP

**Figure 5:**
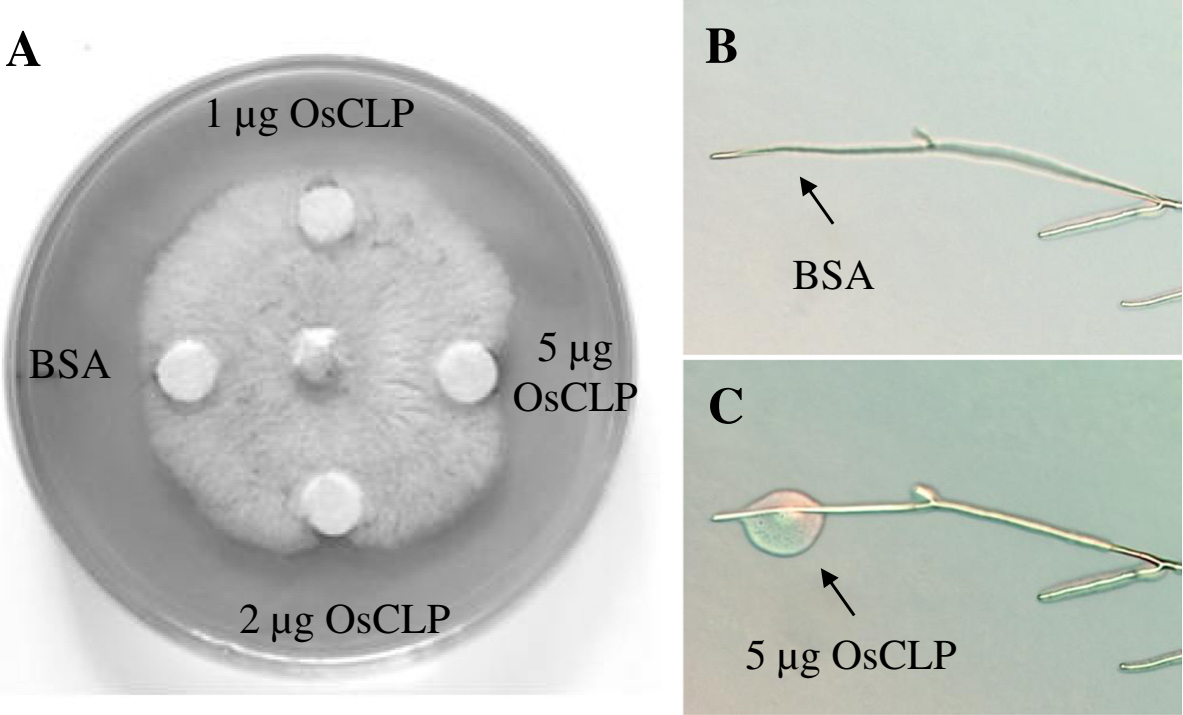
**Antifungal activity of OsCLP protein. A**, growth inhibition of *R. solani* by OsCLP protein. One, two, or five micrograms of purified OsCLP protein were pipetted onto filter paper disks on PDA medium after inoculation with *R. solani*. BSA (5 μg) was used as a negative control. **B**, **C**, microscopic view of growing *R. solani* hyphae treated with 5 μg BSA or 5 μg OsCLP. Arrows indicate regions of cell wall degradation.

The antifungal activity of OsCLP protein was evaluated in the presence of a pathogen. Purified OsCLP protein is unstable to maintain inhibitory activity against slow grower *M. oryzae.* We therefore selected *Rhizoctonia solani*, which grows more quickly than *M. oryzae*, to analyze the biochemical activity of OsCLP. In this experiment, we applied 1, 2, or 5 μg of purified OsCLP protein to *R. solani*. Treatment with 5 μg BSA (negative control) or 1 μg/ml OsCLP protein showed little inhibitory activity on the growth of *R. solani* (Figure 5A). When treated with 2 or 5 μg OsCLP protein, however, the growth of *R. solani* was inhibited (Figure 5A). Therefore, OsCLP displays antifungal activity in a dose-dependent manner.

A close-up view of fungal hyphae after treatment with OsCLP protein was observed through a light microscope to investigate how OsCLP protein suppresses *R. solani* growth. The release of cytosolic contents occurred within 10 minutes of OsCLP treatment (Figure 5B). These data suggest that OsCLP might exhibit strong biochemical activity to dissolve fungal cell walls.

## Conclusions

**Figure 6:**
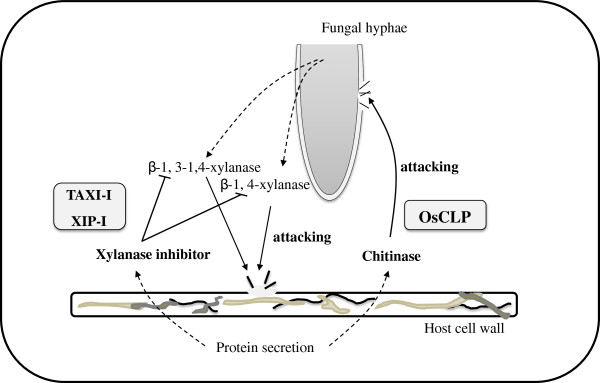
**Proposed model for TAXI-type OsCLP activity during pathogen attack.** Two major ways that the host can suppress fungal infection are indicated. To protect the host from cell-wall degrading enzymes (xylanases) secreted from the fungi, the host secretes enzyme inhibitors (xylanase inhibitors) to suppress biochemical activities. These defense-related proteins contain biochemical activity (chitinase) that is activated by pathogen infection and secreted to directly attack the fungi, resulting in protection of the host cells.

Previous studies have suggested that XIPs are associated with defense, as they play a significant role in protecting host plants from pathogen attack [11]. The amount of OsCLP released in response to pathogen infection increased at both the transcriptional and translational levels during incompatible interactions, suggesting that *OsCLP* is associated with plant defense mechanisms. However, in strong contrast to TAXI-I and XIP-I type inhibitors, recombinant OsCLP protein did not exhibit xylanase inhibitor activity against endo-1,4-β-xylanases secreted from other fungi. Instead, significant chitinase activity was detected in our experiment. This xylanase inhibitor-like chitinase protein inhibited the growth of the fungal pathogen *R. solani*. Fungal cell wall structures were dissolved in the presence of OsCLP. We therefore suggest a new model for OsCLP activity: OsCLP expression is rapidly induced, and this protein is secreted from the host cell, in response to pathogen attack. The protein then attacks the pathogen by degrading the pathogen’s cell wall (Figure 6).

## Methods

### Plant and fungal growth

Rice (cv. Jinheung) seedlings in the 4–5 leaf stage that were grown under natural light in a greenhouse at 28°C were used for whole-plant inoculations with the blast fungus *M. oryzae*. For fungal inoculation of rice leaves, a conidial suspension (1 × 10^5^ conidia/mL) of *M. oryzae* race KJ401 and KJ301 (incompatible and compatible to cv. Jinheung, respectively) was sprayed onto the leaves using an air sprayer. The inoculated plants were kept in a humidity chamber at 28°C and harvested at defined time periods.

### Isolation and cloning of *OsCLP*

The *OsCLP* coding sequence was amplified from a rice cDNA library, which was synthesized from rice leaves inoculated with *M. oryzae* race KJ401 (incompatible cv. Jinheung), using PCR with specific primer sets: Forward, 5'-GGATCCATGTCGCTCCACCTCCTCCTC-3'; Reverse, 5'-AAGCTTCTAGGCGGCGAGAGTGAAGT-3', containing *Bam*HI and *Hin*dIII restriction enzyme sites (underlined). The amplified fragment was gel purified (PCR Clean-up System, Promega, Madison, WI, USA) and cloned into a pGEM-T easy vector (Promega). The product was digested with *Bam*HI and *Hin*dIII and sub-cloned into a pQE30 expression vector. The recombinant plasmid was then transformed into *E. coli* (strain BL21 DE3.0) for protein purification.

### Expression and purification of OsCLP

The *E. coli* cells were grown in LB medium containing 50 μg/ml of ampicillin until OD_600_ was 0.6. IPTG, at a final concentration of 0.1 mM, was added for OsCLP protein induction. *E. coli* cells were cultured at 18°C for 3 days with shaking at 100 × *g* and harvested by centrifugation at 3,500 × *g* for 10 min at 4°C. The pellet was resuspended in lysis buffer (50 mM NaH_2_PO_4_, 300 mM NaCl, 10 mM imidazole, pH 8.0), followed by sonication of the lysed cells and centrifugation at 10,000 × *g* for 20 min. The supernatant was collected and incubated with Ni^2+^- affinity resins at 4°C for 2 h. The collected resins were washed with 5 volumes of washing buffer (50 mM NaH_2_PO_4_, 300 mM NaCl, 20 mM imidazole, pH 8.0), and then eluted with elution buffer (50 mM NaH_2_PO_4_, 300 mM NaCl, 250 mM imidazole, pH 8.0). The eluted protein was stored at −20°C until further analysis. The purified OsCLP recombinant protein containing 6 × His-tagged was used to raise an antibody in rabbit and for the biochemical assay.

### Semi-quantitative RT-PCR

Total RNAs isolated from rice tissue and leaves infected with fungus were extracted using a Plant Mini RNA Kit (Qiagen, Valencia, CA, USA). cDNA was synthesized using a SuperScript III First-Strand Synthesis System (Invitrogen, Madison, WI, USA). Primers were designed to generate PCR products of 300–500 bp, and actin transcript was used as an internal control to normalize the concentration of cDNA in each sample. PCR was carried out using gene-specific primer pairs: *OsCLP* forward, 5'-GGATCTCCCCGGATACTTCA-3'; *OsCLP* reverse 5'-TTCTCCATCTGGAACCCTCC-3'; actin forward, 5'-AGGAATGGAAGCTGCGGGTAT-3'; actin reverse, 5'-GCAGGAGGACGGCGATAACA-3'. The PCR products were separated on a 1% agarose gel stained with ethidium bromide.

### Western blot analysis

Total soluble protein (20 μg) was separated by 12% SDS-PAGE and transferred to a PVDF membrane using a semidry electrophoretic apparatus (Hoefer, Holliston, MA). The blotted membrane was blocked for 4 h at room temperature in 1× TTBS buffer (50 mM Tris–HCl, pH 8.2, 0.1% (v/v) Tween 20, and 150 mM NaCl) with 7% skim milk, followed by incubation for 2 h after the addition of 1/1000 purified OsCLP primary antibody. The membrane was washed three times with 1× TTBS for 15 min each time. A secondary anti-rabbit IgG antibody conjugated with horseradish peroxidase and diluted 1:10,000 in 1× TTBS was used for immunodetection. The antigen-antibody interaction was carried out for 2 h, and cross-reacting proteins were detected by ECL (Perkin Elmer Life Sciences, Boston, MA) using an LAS 4000 imaging system (Fujifilm, Japan).

### Xylanase inhibition assay

Standard xylanases from *A. pullulans* and *T. viride* were purchased from Sigma-Aldrich corporation. A xylanase inhibition assay for OsCLP was performed in a 1.5% LB agar plate containing 1% (w/v) birchwood xylan substrate. Ten micromoles of xylanase enzyme alone, boiled xylanase alone, xylanase coupled with 10 μg OsCLP protein, xylanase coupled with xylanase inhibitor (15 mM *N*-bromosuccinimide, Sigma), and xylanase enzyme coupled with 5 μg BSA were incubated in 1 × PBS buffer (pH 8.0) for 1 h at 30°C and pipetted onto separate 0.5 mm filter disks on the LB agar xylan plate. The plate was incubated in the dark for 24 h at 30°C, followed by staining with 1% Congo red solution for 15 min and destaining with 1 M NaCl. The level of xylanase activity was measured with an LAS 4000 imaging system (Fujifilm, Japan) by detecting degraded circles of xylan. All statistical analyses were performed using Student’s t test (*P* < 0.05).

### In-gel chitinase activity assay

Chitinase activity was assayed with glycol chitin as a substrate using previously described methods [26]. Proteins were separated on a 12.5% SDS-PAGE gel containing 0.01% glycol chitin substrate. After incubating the gel in 100 mM sodium acetate buffer (pH 5.0) containing 1% (v/v) Triton X-100 at 37°C for 2 h, the gel was stained with 500 mM Tris–HCl (pH 8.9), 0.01% (w/v) Fluorescent Brightener 28 (Sigma-Aldrich) solution for 10 min. Fluorescent signals were detected under UV using an LAS 4000 system after destaining the gel in distilled water for 20 min.

### Antifungal activity of OsCLP

The fungal pathogen *Rhizoctonia solani* was used for the hyphal growth inhibition assay of the OsCLP protein [27]. *R. solani* was inoculated on a potato dextrose agar (PDA) plate and cultured at 28°C in the dark. Filter paper was placed onto the PDA plate 5 mm away from the growing hyphae. After placing OsCLP protein or BSA onto the filter paper, the PDA plate was incubated for 4 h at 28°C. Fungal hyphal growth was measured with a digital camera. For a close-up view of degrading hyphae, the growing hyphae on the PDA plate were covered with enzyme and observed until the release of cytosolic contents was detected under a microscope (AX70TRF, Olympus, Japan).

## Competing interests

The authors declare that they have no competing interests.

## Authors’ contributions

KYK designed the experiments. JW and YW carried out the experiments. JW, YW, SGK, and STK analyzed the data. JW, YW, and SGK wrote the paper. SGK and KYK were the advisor and group leader, respectively. All authors read and approved the final version of this manuscript**.**
